# Construction of a prediction model for pulmonary infection and its risk factors in Intensive Care Unit patients

**DOI:** 10.12669/pjms.40.6.9307

**Published:** 2024-07

**Authors:** Weilei Dai, Ting Zhong, Feng Chen, Miaomiao Shen, Liya Zhu

**Affiliations:** 1 Weilei Dai Department of Nursing, Jiaxing Hospital of Traditional Chinese Medicine Jiaxing, Zhejiang Province 314001, P.R. China; 2 Ting Zhong Department of ICU, Jiaxing Hospital of Traditional Chinese Medicine Jiaxing, Zhejiang Province 314001, P.R. China; 3 Feng Chen Department of ICU, Jiaxing Hospital of Traditional Chinese Medicine Jiaxing, Zhejiang Province 314001, P.R. China; 4 Miaomaio Shen Department of Information Center, Jiaxing Hospital of Traditional Chinese Medicine Jiaxing, Zhejiang Province 314001, P.R. China; 5 Liya Zhu Department of ICU, Jiaxing Hospital of Traditional Chinese Medicine Jiaxing, Zhejiang Province 314001, P.R. China

**Keywords:** Intensive care unit, Pulmonary infection, Nomogram, Risk factor

## Abstract

**Objective:**

To identify independent risk factors of pulmonary infection in intensive care unit (ICU) patients, and to construct a prediction model.

**Methods:**

Medical data of 398 patients treated in the ICU of Jiaxing Hospital of Traditional Chinese Medicine from January 2019 to January 2023 were analyzed. Univariate and multivariate logistic regression analyses were used to identify independent risk factors for pulmonary infection in ICU patients. R software was used to construct a nomogram prediction model, and the prediction model was internally validated using computer simulation bootstrap method. Predictive value of the model was analyzed using the receiver operating characteristic (ROC) curve.

**Results:**

A total of 97 ICU patients (24.37%) developed pulmonary infection. Age, ICU stay time, invasive operation, diabetes, duration of mechanical ventilation, and state of consciousness were all identified as risk factors for pulmonary infection. The calibration curve of the constructed nomogram prediction model showed a good consistency between the predicted value of the model and the actual observed value. ROC curve analysis showed that the area under the curve (AUC) of the model was 0.784 (95% CI: 0.731-0.837), indicating a certain predictive value.

**Conclusions:**

Age, length of stay in ICU, invasive operation, diabetes, duration of mechanical ventilation, and state of consciousness are risk factors for pulmonary infection in ICU patients. The nomogram prediction model constructed based on the above risk factors has shown a good predictive value.

## INTRODUCTION

Pulmonary infection, a common complication in intensive care unit (ICU) patients, usually occurs after the first 24 hours of ventilator support.^1^,^2^ Studies show that among patients receiving treatment in the ICU, the mortality rate caused by pulmonary infections can reach 9% to 50%.^3^ Moreover, the concurrent pulmonary infection in ICU patients may prolong their ICU treatment time, which not only increases patient mortality but is also associated with a substantial burden on the healthcare system.^4^,^5^ Compared to patients without pulmonary infection, the average medical expenses of patients with pulmonary infection can increase by 2-10 times.^3^–^5^ Therefore, there is constant need to further improve the prevention of pulmonary infection in ICU patients and to identify relevant risk factors.

In recent years, numerous studies have focused on the various risk factors of lung infection in ICU patients but with controversial results.^6^–^9^ The variability between the studies may be attributed to factors such as the variability in the baseline characteristics of the patient cohorts, different pathogenic bacteria strains, treatment situation, etc.^7^ Moreover, most studies focus on predicting prolonged ICU stays^10^ or in-hospital mortality in ICU patients^11^,^12^, but few studies have constructed prediction models for pulmonary infection in ICU patients.

Current study retrospectively selected medical records of 398 patients admitted to the ICU of our hospital in the past four years. The main goal of the study was to identify independent risk factors of pulmonary infection in intensive care unit (ICU) patients, and to construct a nomogram prediction model that can be used for the prevention and control of pulmonary infections this cohort in clinical practice.

## METHODS

Clinical data of 398 patients admitted to the ICU of Jiaxing Hospital of Traditional from January 2019 to January 2023 were retrospectively analyzed. Baseline data of patients were collected, and univariate and multivariate binary logistic regression analyses were done.

### Inclusion criteria:

- Staying in the ICU for more than 48 hours.
- No pulmonary infection or pneumonia before admission to the ICU.
- Patient underwent mechanical ventilation through tracheal intubation.
- Complete medical record information.

### Exclusion criteria:

- Patients who developed pulmonary infection before admission to ICU.
- Patients with a long-term history of taking hormones and immunosuppressants.
- Abandoning treatment midway.
- Pregnant female patients.
- Patients with concomitant mental illness.

### Ethical Approval

The Medical Ethics Committee of our hospital approved this study (No. SL-2023-0069, Date: June 19, 2023).

### Diagnostic criteria for pulmonary infection

13 Patients who met two or more of the following conditions were diagnosed with pulmonary infection:

- Body temperature higher than 38°C and lasted for 4-6 days;
- White blood cell count >10×10^9^/L;
- Three consecutive sputum examinations showed the presence of pathogenic bacteria and increased sputum viscosity;
- Computed tomography examination of the lungs showed the presence of inflammatory lesions.

### Data collection

We retrospectively collected the clinical data of the patients from the department electronic case system, including demographic characteristics (age, gender, BMI, education level), disease type (diseases of nervous system, cardiovascular disease, respiratory diseases, major operation, other), length of stay in ICU, mechanical ventilation duration, invasive surgery (yes, no), history of diabetes (yes, no), history of hypertension (yes, no), state of consciousness (sober, coma), antibacterial drugs use (yes, no), and hormone use (yes, no).

### Statistical analysis

SPSS22.0 and R software version 4.0.0. were used. Measurement data that followed a normal distribution were represented by (*χ̅*±*S*), and independent sample t-test was used for inter group comparisons. Data that did not meet the normal distribution were represented by M (IQR), and inter group comparisons were conducted using Mann Whitney U test. The counting data were represented by n (%), and the comparison between groups was performed using chi square test. A binary logistic regression model was used to analyze the risk factors of pulmonary infection, and a nomogram prediction model was constructed using the “rms” package of the R software. ROC curve was established to analyze the predictive performance of the nomogram model. *P*<0.05 was statistically significant.

## RESULTS

A total of 398 patients were included in this study, of which 97 (24.37%) developed pulmonary infections. There was no statistically significant difference in gender, BMI, educational level, disease type, concomitant hypertension, antibiotic use, and hormone use between infected and non-infected patients (*P*>0.05). However, there were significant differences in age, ICU stay time, invasive operation, diabetes, mechanical ventilation, and state of consciousness (*P*<0.05) (Table-I).

**Table-I:** Comparison of clinical data between the two groups.

	No pulmonary infection (n=301)	Pulmonary infection (n=97)	χ^2^/t/z	P
Age [n (%)]			44.208	0.000
<60 years old	205(68.1)	29(29.9)		
≥60 years old	96(31.9)	68(70.1)		
Gender [n (%)]			0.284	0.594
Male	152(50.5)	52(53.6)		
Female	149(49.5)	45(46.4)		
BMI (kg/m2)	26.68±2.26	26.59±2.34	0.304	0.762
Education level [n (%)]			1.365	0.505
Junior high school and below	141(46.8)	45(46.4)		
Technical secondary school/high school	118(39.2)	34(35.1)		
College degree or above	42(14.0)	18(18.6)		
Disease type [n (%)]			1.416	0.841
Diseases of nervous system	92(30.6)	28(28.9)		
Cardiovascular disease	87(28.9)	27(27.8)		
Respiratory diseases	58(19.3)	21(21.6)		
Major operation	55(18.3)	16(16.5)		
Other	9(3.0)	5(5.2)		
Length of stay in ICU (day)	9(7,11)	11(8,14)	-5.43	0.000
Invasive surgery [n (%)]			5.754	0.016
No	199(66.1)	51(52.6)		
Yes	102(33.9)	46(47.4)		
History of diabetes [n (%)]			7.657	0.006
No	216(71.8)	55(56.7)		
Yes	85(28.2)	42(43.3)		
History of hypertension [n (%)]			0.147	0.702
No	211(70.1)	66(68)		
Yes	90(29.9)	31(32)		
Mechanical ventilation duration (day)	4(3,5)	7(6,7)	-10.975	0.000
State of consciousness [n (%)]			11.729	0.001
Sober	123(40.9)	21(21.6)		
Coma	178(59.1)	76(78.4)		
Antibacterial drugs use [n (%)]			1.149	0.284
No	186(61.8)	54(55.7)		
Yes	115(38.2)	43(44.3)		
Hormone usage [n (%)]			0.001	0.971
No	193(64.1)	62(63.9)		
Yes	108(35.9)	35(36.1)		

Multivariate logistic regression analysis was carried out and showed that age, ICU stay time, invasive operation, diabetes, duration of mechanical ventilation, and state of consciousness were all risk factors for lung infection (Table-II).

**Table-II:** Results of multivariate Logistic regression analysis

Variable	B	S.E.	Wald	P	OR	95%CI	

						Lower limit	Upper limit
Age	1.533	0.272	31.850	0.000	4.630	2.719	7.884
Length of stay in ICU	1.385	0.392	12.457	0.000	3.994	1.851	8.616
Invasive surgery	0.718	0.268	7.169	0.007	2.051	1.212	3.469
History of diabetes	0.684	0.273	6.276	0.012	1.982	1.161	3.385
Mechanical ventilation duration	1.701	0.753	5.108	0.024	5.480	1.254	23.958
State of consciousness	0.710	0.299	5.654	0.017	2.034	1.133	3.651

**Fig.1:**
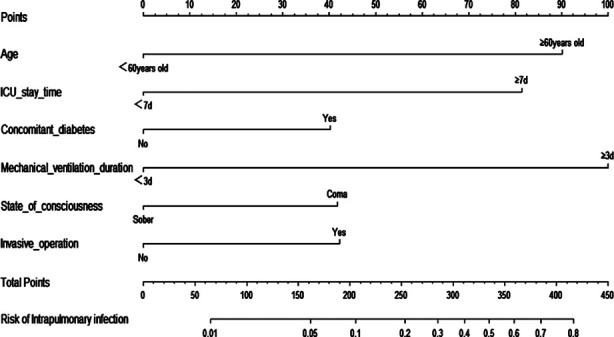
Nomogram Prediction Model.

**Fig.2:**
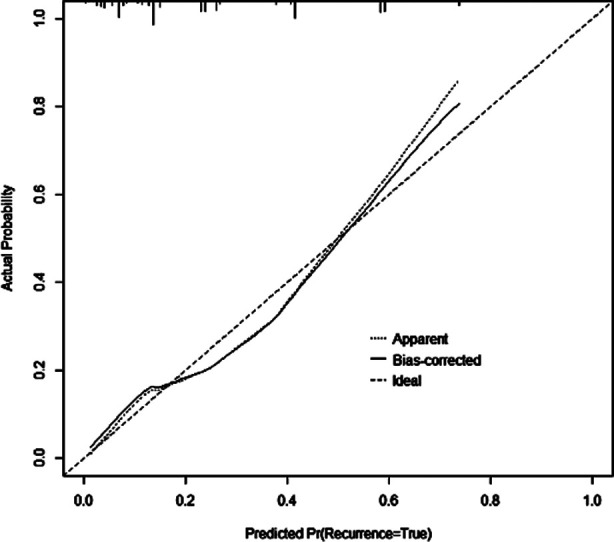
Calibration Curve of the Nomogram.

**Fig.3:**
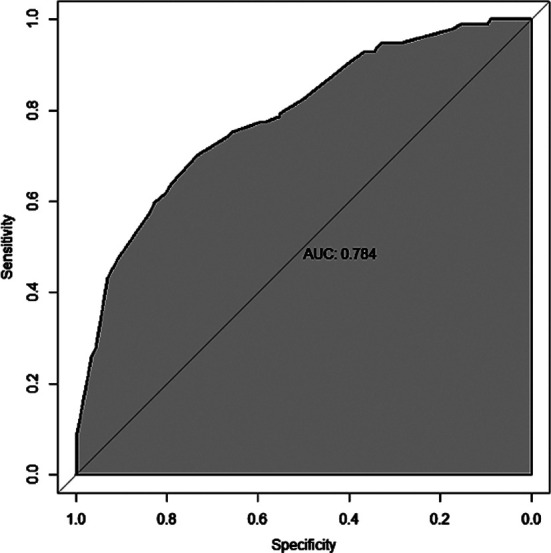
ROC curve of the Nomogram.

Based on the six risk factors mentioned above, a nomogram prediction model was constructed using the “RMS” package of R software 4.0.0 (Fig.1). The calibration curve of the nomogram prediction model for predicting pulmonary infection in ICU patients showed good consistency in this cohort (Fig.2). Internal validation of the prediction model was carried out using the bootstrap method and indicated that the prediction model basically fits the ideal model. The AUC of the ROC curve in the nomogram prediction model was 0.784 (95% CI: 0.731-0.837), indicating a certain predictive value. When the optimal cut off value was selected, the sensitivity and specificity of the model were 70.1% and 73.4%, respectively, indicating that the prediction model is effective (Fig.3).

## DISCUSSION

This study reported that the incidence of pulmonary infections in the cohort of 398 ICU patients was 24.37%. Further analysis showed that age, ICU stay time, invasive operations, diabetes, duration of mechanical ventilation, and state of consciousness were all independent risk factors of pulmonary infection in ICU patients. Prediction model, constructed using these factors, has a good predictive value.

Pulmonary infection is one of the most common hospital-acquired infections.^7^,^14^ Numerous studies have shown that ICU patients often have suppressed immune defenses, reduced ability to resist pathogens, and are highly susceptible to pulmonary infections, which further worsens the condition of patients and prolongs their stay in the ICU, thus forming a vicious cycle.^7^,^15^ Current research indicates that the risk of acquiring in-hospital pulmonary infection is mainly related to the patient’s own health and functional status.^15^,^16^ Our study showed that age was one of the independent risk factors of pulmonary infection in this population of patients. Our results are consistent with the study of Li et al.,^17^ which showed that higher age correlates with a higher risk of developing lung infections. This may be explained by the fact that elderly patients often have problems with small airway closure and lung atelectasis.^18^ Respiratory movement function in these patients is reduced, sputum excretion is difficult, and the risk of respiratory pathogen invasion and reproduction is increased. Additionally, the overall decline in the function of various vital organs and frailty may lead to a decrease in the ability of elderly patients to resist pathogen infections, resulting in an increase in the incidence of lung infections.^17^,^18^

Length of stay in ICU was also associated with the increased risk of pulmonary infections in our cohort, which is in agreement with the study by Lan et al.^19^ Prolonged bedrest leads to the limited lung expansion and accumulation of respiratory secretions, providing good conditions for pathogen colonization and causing lung infections.^19^

We showed that the risk of pulmonary infections was higher in ICU patients after the invasive surgery. This is similar to the study by Gil et al.^20^ We may speculate that postoperative ICU patients often need to undergo invasive procedures such as tracheal intubation and deep vein intubation, which can reduce the body’s defense function and increase the risk of pulmonary infection.

The rate of lung infections was higher in patients with diabetes. Most patients with diabetes have abnormal protein and fat metabolism, which can lead to increased body consumption, decreased muscle strength, respiratory muscle atrophy, reduced deep breathing function and cough ability, thus increasing the risk of lung infection.^21^

Mechanical ventilation duration was an independent risk factor of pulmonary infection in our study. Similarly, Zhang et al.^22^ showed that mechanical ventilation time ≥3 days in ICU patients is a risk factor for pulmonary infection. ICU patients often require tracheal intubation during mechanical ventilation, which can disrupt the protective function of the upper respiratory tract and provide good conditions for pathogen invasion. The longer the mechanical ventilation time, the greater the irritation to the respiratory tract, and the more severe the mechanical damage to the airway mucosa. The function of clearing secretions in the respiratory tract is reduced, which is conducive to the proliferation of pathogenic bacteria.^23^,^24^ Moreover, patients with systemic ventilation often experience catheter intolerance and restlessness, requiring corresponding sedative and analgesic drugs.^22^ This may have an inhibitory effect on the respiratory control center and protective reflexes, leading to a decrease in respiratory function and causing reflux and aspiration of gastric contents, leading to pulmonary infection.^24^,^25^

Our results showed that the state of consciousness correlated with the risk of pulmonary infections. Patients with altered consciousness may experience swallowing loss, and weak or absent cough reflex. Additionally, overstimulated vagus nerve in such patients can lead to bronchospasm and accumulation of secretion, thus promoting the colonization of pathogenic bacteria and lung infection.^24^,^26^

In recent years, nomogram has been widely used for medical research. The current study also found that the nomogram prediction model constructed based on the identified risk factors has shown a good predictive value, which is generally consistent with the findings by Wu et al^27^ and Gan et al^28^. The nomogram model may facilitate early prediction and interventions for pulmonary infections in clinical practice.

### Limitations

This is a single center retrospective study with a sample size of only 398 cases, which may be biased. In addition, there may be significant differences in lung infections caused by different disease types, and many potential risk factors that may have not been considered in this study. Furthermore, only a few variables were selected for regression analysis, which may lead to subjectivity and one-sidedness in the results.

## CONCLUSION

Age, length of stay in ICU, invasive procedures, diabetes, duration of mechanical ventilation, and state of consciousness were identified as independent risk factors of pulmonary infections in ICU patients. The nomogram prediction model constructed based on the above factors has shown a good predictive value and may be used in clinical practice.

### Authors’ contributions:

**WD** conceived and designed the study.

**TZ, FC, MS and LZ** collected the data and performed the analysis.

**WD** was involved in the writing of the manuscript and is responsible for the integrity of the study.

All authors have read and approved the final manuscript.
